# Thymic stromal lymphopoietin *(TSLP) *is associated with allergic rhinitis in children with asthma

**DOI:** 10.1186/1476-7961-9-1

**Published:** 2011-01-18

**Authors:** Supinda Bunyavanich, Erik Melen, Jemma B Wilk, Mark Granada, Manuel E Soto-Quiros, Lydiana Avila, Jessica Lasky-Su, Gary M Hunninghake, Magnus Wickman, Göran Pershagen, George T O'Connor, Scott T Weiss, Juan C Celedόn

**Affiliations:** 1 Channing Laboratory, Department of Medicine, Brigham and Women's Hospital, Boston, Massachusetts, USA; 2 Division of Rheumatology, Immunology and Allergy, Department of Medicine, Brigham and Women's Hospital, Boston, Massachusetts, USA; 3 Division of Pulmonary and Critical Care Medicine, Department of Medicine, Brigham and Women's Hospital, Boston, Massachusetts, USA; 4 Center for Genomic Medicine, Department of Medicine, Brigham and Women's Hospital, Boston, Massachusetts, USA; 5 Harvard Medical School, Boston, Massachusetts, USA; 6 Institute of Environmental Medicine, Karolinska Institutet, Stockholm, Sweden; 7 Astrid Lindgren Children's Hospital, Karolinska University Hospital, Stockholm, Sweden; 8 Sachs Children's Hospital, Stockholm, Sweden; 9 Department of Neurology, Department of Medicine, Boston University School of Medicine, Boston, Massachusetts, USA; 10 Pulmonary Medicine, Department of Medicine, Boston University School of Medicine, Boston, Massachusetts, USA; 11 The National Heart, Lung, and Blood Institute's Framingham Heart Study, Framingham, Massachusetts, USA; 12 Division of Pediatric Pulmonology, Hospital Nacional de Niños, San José, Costa Rica; 13 Division of Pulmonary Medicine, Allergy, and Immunology, Children's Hospital of Pittsburgh of the University of Pittsburgh Medical Center, Pittsburgh, Pennsylvania, USA; 14 Department of Internal Medicine, University of Pittsburgh School of Medicine, Pittsburgh, Pennsylvania, USA; 15 Department of Human Genetics, University of Pittsburgh Graduate School of Public Health, Pittsburgh, Pennsylvania, USA

## Abstract

**Background:**

Allergic rhinitis (AR) affects up to 80% of children with asthma and increases asthma severity. Thymic stromal lymphopoietin (TSLP) is a key mediator of allergic inflammation. The role of the TSLP gene (*TSLP*) in the pathogenesis of AR has not been studied.

**Objective:**

To test for associations between variants in *TSLP*, *TSLP*-related genes, and AR in children with asthma.

**Methods:**

We genotyped 15 single nucleotide polymorphisms (SNPs) in *TSLP, OX40L, IL7R*, and *RXRα *in three independent cohorts: 592 asthmatic Costa Rican children and their parents, 422 nuclear families of North American children with asthma, and 239 Swedish children with asthma. We tested for associations between these SNPs and AR. As we previously reported sex-specific effects for *TSLP*, we performed overall and sex-stratified analyses. We additionally performed secondary analyses for gene-by-gene interactions.

**Results:**

Across the three cohorts, the T allele of *TSLP *SNP rs1837253 was undertransmitted in boys with AR and asthma as compared to boys with asthma alone. The SNP was associated with reduced odds for AR (odds ratios ranging from 0.56 to 0.63, with corresponding Fisher's combined P value of 1.2 × 10^-4^). Our findings were significant after accounting for multiple comparisons. SNPs in *OX40L, IL7R*, and *RXRα *were not consistently associated with AR in children with asthma. There were nominally significant interactions between gene pairs.

**Conclusions:**

*TSLP *SNP rs1837253 is associated with reduced odds for AR in boys with asthma. Our findings support a role for *TSLP *in the pathogenesis of AR in children with asthma.

## Introduction

Allergic rhinitis (AR) is a common chronic disease, affecting 10-30% of adults and 40% of children [1]. Characterized by nasal congestion, itching, rhinorrhea, and sneezing, AR decreases school and work productivity. AR is a risk factor for asthma exacerbations and asthma-related hospitalization [2,3]. Up to 80% of children with asthma have AR [4], and treatment of comorbid AR reduces the odds of asthma-related healthcare by up to 80% [5]. A better understanding of the pathophysiology of AR could decrease morbidity in asthmatics and in children overall.

**Figure 1:**
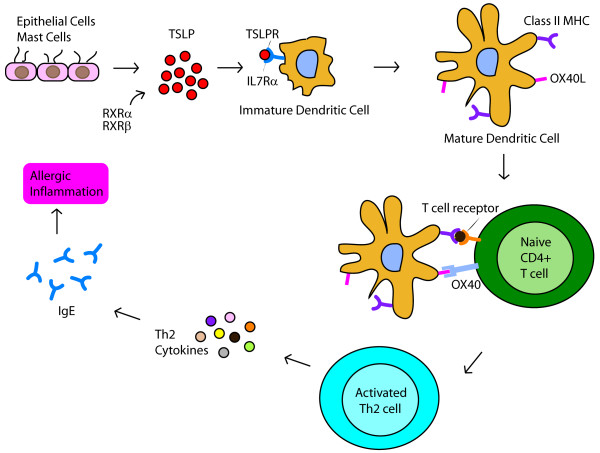
**TSLP is a key mediator of allergic inflammation**. TSLP binds to a heterodimeric receptor of TSLPR and IL7Rα chains. Its induction of Th2 mediated inflammation requires costimulation by OX40L binding to OX40. RXRα and RXRβ act as transcriptional regulators.

The role of thymic stromal lymphopoietin (TSLP) in the pathogenesis of AR has not been extensively studied. TSLP is an interleukin (IL)-7-like cytokine that triggers dendritic cells and mast cells to induce T helper (Th)2 inflammatory responses (Figure 1) [6,7]. The gene for TSLP (*TSLP*) is expressed by epithelial cells of the lung, skin and gut [8]. In humans, *TSLP *has been linked to the pathogenesis of asthma [9-11], atopic dermatitis [6], and eosinophilic esophagitis [12]. A few *in vitro *and murine studies with small sample size have examined *TSLP *expression in allergic rhinitis (AR) [13-16]. To date, there have been no genetic association studies of *TSLP *and AR.

The effects of TSLP are influenced by its heterodimeric receptor, costimulatory molecules, transcriptional regulators, and other cytokines (Figure 1). Therefore, we were also interested in examining the association between AR and genes related to *TSLP*, including *OX40L, IL7R*, and *RXRα*. Expressed by antigen-presenting cells, *OX40L *is an essential costimulatory mediator of TSLP-mediated Th2 responses [17,18]. Blockade of OX40L inhibits TSLP-driven Th2 inflammatory cell infiltration, cytokine secretion, and IgE production in mouse lung and skin [19]. The receptor for TSLP is a heterodimeric complex composed of TSLPR and IL7Rα chains [20]. The TSLPR chain binds to TSLP at low affinity but its combination with the IL7Rα chain results in high-affinity binding and STAT5 activation [21,22]. The IL7Rα chain is encoded by *IL7R*. The nuclear receptors retinoid × receptor (RXR)α and RXRβ act as transcriptional repressors that inhibit *TSLP *gene expression in mouse skin keratinocyte models of atopic dermatitis [23].

In this study, we report an analysis of association between single nucleotide polymorphisms (SNPs) in *TSLP, OX40L, IL7R*, and *RXRα *and AR in three independent studies of children with asthma from Costa Rica, North America, and Sweden. We chose to perform this study in children with asthma because AR causes disproportionately high morbidity in children with asthma, and AR is up to four times more prevalent in children with asthma [24]. We found that a SNP in *TSLP *was associated with reduced odds for AR in boys across the three cohorts of children with asthma.

## Methods

### Ethics Statement

Written informed consent was obtained from study participants and from parents of children in the cohorts. The institutional review boards of Brigham & Women's Hospital, CAMP Study Centers, and Karolinska Institutet approved the study protocols.

### Study Populations

#### The Genetics of Asthma in Costa Rica

The Genetics of Asthma in Costa Rica study includes 616 children ages 6-14 years with asthma who were recruited between February 2001 and March 2006 [4]. This population is a genetic isolate of mixed Spanish and Amerindian descent with one of the world's highest rates of asthma (27.4% of children aged 6-7 years [25]). Questionnaires were sent to the parents of 13,125 schoolchildren enrolled in 113 schools in Costa Rica. Of the 7,282 children whose parents returned questionnaires, 2,714 had asthma (defined as physician-diagnosed asthma and ≥2 respiratory symptoms or recurrent asthma attacks in the past year). Of these 2,714 children, 616 had high probability of having ≥6 great-grandparents born in the Central Valley of Costa Rica (to ensure descent from the founder population), and were willing to participate along with their parents. Of these 616 parent-child trios, 24 were excluded because of inadequate DNA quality, leaving 592 trios for genotyping and analysis.

#### Childhood Asthma Management Program (CAMP)

CAMP is a multicenter North American clinical trial designed to investigate the long-term effects of inhaled anti-inflammatory medications in children with mild to moderate asthma [26]. Participating children had asthma defined by symptoms greater than 2 times per week, use of an inhaled bronchodilator at least twice weekly or use of daily medication for asthma, and increased airway responsiveness to methacholine (PC_20 _≤ 12.5 mg/ml). Children with severe asthma or other clinically significant medical conditions were excluded. Of the 1041 children enrolled in the original clinical trial, 968 children and 1518 of their parents contributed DNA samples to the CAMP Genetics Ancillary Study [27]. Selection criteria for genome wide association study (GWAS) genotyping were (a) self-described non-Hispanic white ethnicity and (b) availability of sufficient DNA for microarray hybridization; 422 children and their parents met these criteria.

#### Children, Allergy, Milieu, Stockholm, Epidemiological Survey (BAMSE)

BAMSE is a birth cohort study of allergy and environment. 4089 newborn infants were recruited between 1994 and 1996 from central and northwestern parts of Stockholm, Sweden [28]. At eight years of age, all BAMSE children were invited for clinical testing, and blood samples were obtained from 2,480 children. DNA was extracted from 2,033 samples after exclusion of samples with too little blood, lack of questionnaire data, or if parental consent to genetic analysis of the sample was not obtained. All children with a doctor's diagnosis of asthma ever (n = 251) underwent GWAS genotyping [29].

### Phenotyping

Phenotypic data were collected from each participant in Costa Rica at study entry, in CAMP at randomization, and in BAMSE at one, two, four, and eight years of age. AR was defined as naso-ocular symptoms apart from colds in the past 12 months and ≥1 positive skin test reaction (STR) to allergens in Costa Rica and CAMP, and as naso-ocular symptoms in the past 12 months and ≥1 positive allergen-specific IgE (Phadiatop^®^, Phadia AB, Uppsala, Sweden) at eight years in BAMSE. These definitions for AR are consistent with Allergic Rhinitis and its Impact on Asthma (ARIA) 2008 guidelines [30]. We chose not to use a physician's diagnosis to define AR given greater variability associated with this definition. We compared allergen-sensitized AR and physician-diagnosed AR in Costa Rica in a previous study [4].

### Genotyping

Using data from European Americans (CEU) in the International HapMap project [31], we applied a linkage-disequilibrium (LD)-tagging algorithm (minor allele frequency ≥ 5% and r^2 ^≥ 0.8) to identify common variation in *TSLP, OX40L, IL7R, RXRα *and their 10 kb flanks. LD maps were plotted using Haploview [32]. We considered additional SNPs in *TSLP *to evaluate reported functional variation (rs3806933) [33] and those highlighted in previous studies of asthma (rs1837253) [34]. A total of 21 SNPs were chosen for genotyping in Costa Rican subjects and their parents using the Sequenom iPLEX platform (Sequenom, San Diego, CA) including 9 SNPs for *TSLP *and 4 SNPs each for *OX40L, IL7R*, and *RXRα*. We chose Costa Rica as our population for initial findings because of greater power to detect associations in this cohort relative to CAMP and BAMSE (Table 1). All power calculations were performed using Quanto v.1.2.4. (University of Southern California, Los Angeles, CA).

**Table 1:** Power calculations for genetic association studies of AR in Costa Rica, CAMP, and BAMSE

	Costa Rica (n = 592)		CAMP (n = 422)		BAMSE (n = 239)	
Allele Frequency	Power to detect OR 1.5	Power to detect OR 1.3	Power to detect OR 1.5	Power to detect OR 1.3	Power to detect OR 1.5	Power to detect OR 1.3
0.10	0.89	0.52	0.77	0.40	0.23	0.12
0.20	0.99	0.75	0.94	0.61	0.36	0.18
0.30	0.997	0.85	0.98	0.71	0.44	0.22
0.40	0.998	0.88	0.98	0.76	0.48	0.24

Genome-wide SNP genotyping for CAMP subjects and their parents was performed on Illumina Human-Hap550 Genotyping BeadChip (Illumina, Inc., San Diego, CA). Genome-wide SNP genotyping for BAMSE subjects was performed on Illumina Human 610-Quad Beadchip (Illumina, Inc., San Diego, CA). Sixteen of the CAMP GWAS SNPs and fifteen of the BAMSE GWAS SNPs overlapped with those genotyped in Costa Rican subjects and their parents. The 15 overlapping SNPs were used to replicate our initial findings (Table 2). Of these 15 SNPs, 3 were in *TSLP *(rs1837253, rs2289276, rs17551370), and 4 each were in *OX40L *(rs1234313, rs10489267, rs10489266, rs1234315), *IL7R *(rs1494555, rs10063294, rs2194225, rs6897932), and *RXRα *(rs11185647, rs12339187, rs11185659, rs10881582). The 6 SNPs that were genotyped in Costa Rica but not on both the GWAS platforms had no significant association with AR in Costa Rica. There were no differences in SNP minor allele frequencies between boys and girls in these cohorts.

**Table 2:** SNPs analyzed in Costa Rica, CAMP, and BAMSE

SNP	Gene	Position	Allele	Allele Frequency		
				**Costa Rica**	**CAMP**	**BAMSE**

rs1837253	*TSLP*	chr5:110429771	T	0.24	0.21	0.27
rs17551370	*TSLP*	chr5:110432084	A	0.10	0.13	0.15
rs2289276	*TSLP*	chr5:110435406	T	0.30	0.27	0.29
rs1234313	*OX40L*	chr1:171432870	A	0.38	0.32	0.34
rs10489267	*OX40L*	chr1:171436775	A	0.14	0.06	0.07
rs10489266	*OX40L*	chr1:171445076	G	0.08	0.11	0.10
rs1234315	*OX40L*	chr1:171445086	T	0.65	0.46	0.48
rs1494555	*IL7R*	chr5:35906947	G	0.47	0.34	0.26
rs6897932	*IL7R*	chr5:35910332	T	0.16	0.24	0.32
rs10063294	*IL7R*	chr5:35913262	G	0.60	0.48	0.42
rs2194225	*IL7R*	chr5:35919561	C	0.37	0.42	0.42
rs11185647	*RXRα*	chr9:136355649	A	0.41	0.29	0.26
rs12339187	*RXRα*	chr9:136369148	G	0.19	0.17	0.17
rs11185659	*RXRα*	chr9:136383204	T	0.22	0.20	0.21
rs10881582	*RXRα*	chr9:136395899	A	0.34	0.23	0.20

CAMP: Childhood Asthma Management Program

BAMSE: Children, Allergy, Milieu, Stockholm, Epidemiological Survey

In all study cohorts, duplicate genotyping was performed on approximately 5% of the sample to assess genotype reproducibility. Genotype quality control was assessed by <1% discordance rate, <5 Mendelian inconsistencies, and genotype completion rates >98% for all loci. All SNPs included in analyses were in Hardy-Weinberg equilibrium (p > 0.01).

Of the 592 Costa Rican child-parent trios genotyped, 6 were excluded from this analysis because of Mendelian inconsistencies, leaving 586 trios. Of the 422 nuclear families in CAMP trios, 25 were excluded from this analysis because of Mendelian inconsistencies (n = 6) or missing >5% of the genotypic data (n = 19), leaving 397 families. 12 children from BAMSE were excluded because of duplicate genotyping or non-European ancestry as determined by admixture mapping using principal components analysis [29], leaving 239 children for this analysis.

### Statistical Analyses

We tested for association between SNPs in *TSLP, OX40L, IL7R, RXRα *and AR in children with asthma. Family-based association analyses were first conducted under an additive genetic model in Costa Rican families using the Pedigree-Based Association Test (PBAT) [35] implemented in Helix Tree v6.4.3 (Golden Helix, Bozeman, MT). An advantage of family-based association testing is that it is robust against population stratification and population admixture [36]. We then replicated our findings from Costa Rica in the CAMP and BAMSE cohorts. Family-based analysis using PBAT was performed in CAMP. In BAMSE, associations between SNPs and AR phenotypes were measured using the Cochran-Armitage trend test in PLINK [37]. As we have previously reported sex-specific effects for *TSLP *on serum total IgE [38] and asthma [11], we also performed sex-stratified analyses in all cohorts. Transmitted to undertransmitted ratios (T:U) and odds ratio estimates for AR phenotypes were obtained using PLINK [37]. To assess for joint evidence of association in the child-based cohorts, P values were combined across Costa Rica, CAMP, and BAMSE with Fisher's combined probability method [39]. Results were considered significant only when consistent associations (i.e. same allele, same direction of genetic effect) were observed in all three populations with a Fisher's combined P value of ≤ 8.0 × 10^-4 ^(0.05/(21*3) to account for multiple testing of 21 SNPs and 3 strata (overall, male, female).

Tests for interaction between SNPs in *TSLP, OX40L, IL7R, RXRα *were additionally performed using PBAT given high interest in potential gene by gene interactions. Based on our power calculations using Quanto v.1.2.4 for gene by gene interactions, we recognized *a priori *that our power to detect such interactions would be insufficient. For example, to detect an interaction between two SNPs each with minor allele frequency 0.40 causing a change in risk of 10%, our sample size would have to be 4229 parent-child trios. We therefore limited our interaction testing to the cohort with most subjects (Costa Rica, with 592 trios) and considered this a secondary, exploratory analysis.

## Results

The phenotypic characteristics of children in the Costa Rica, Childhood Asthma Management Program (CAMP), and Children, Allergy, Milieu, Stockholm, Epidemiological Survey (BAMSE) study cohorts are shown in Table 3. Consistent with the known gender distribution of asthma in childhood [40], all three cohorts had more boys than girls. Children in Costa Rica had the highest prevalence of AR.

**Table 3:** Baseline phenotypic characteristics of children in Costa Rica, CAMP, and BAMSE

Variable	Costa Rica^*^			CAMP^*^			BAMSE		
	**All (n = 592)**	**Boys (n = 351)**	**Girls (n = 241)**	**All (n = 422)**	**Boys (n = 266)**	**Girls (n = 156)**	**All (n = 239)**	**Boys (n = 150)**	**Girls (n = 89)**

**Age-Years**	9.0 (1.8)	9.1 (1.9)	8.9 (1.7)	8.7 (2.1)	8.6 (2.1)	8.9 (2.1)	8.3 (0.5)	8.3 (0.5)	8.3 (0.4)

**STR or allergen-specific IgE - Any Positive**	504 (85.4%)	200 (83.9%)	304 (87.1%)	370 (87.7%)	235 (88.4%)	135 (86.5%)	113 (47.3%)	78 (52%)	35 (39%)

**Serum Total IgE - IU/ml**	697 (867)	755 (911)	611 (792)	1095 (1920)	988 (1567)	1276 (2397)	n/a^†^	n/a^†^	n/a^†^

**Nasal symptoms in past year**	543 (91.7%)	334 (90.3%)	230 (93.5%)	207 (49.1%)	135 (50.8%)	72 (46.2%)	76 (31.9%)	52 (34.9%)	24 (27.0%)

**Allergic Rhinitis**	474 (80.1%)	282 (80.3%)	192 (79.7%)	189 (44.8%)	124 (46.6%)	65 (41.7%)	64 (26.9%)	43 (28.9%)	21 (23.6%)

Values are presented as mean (standard deviation) or number (percentage)

STR = skin test reaction

*Values are for index children in these family-based cohorts

^†^Data not collected in BAMSE

**Figure 2:**
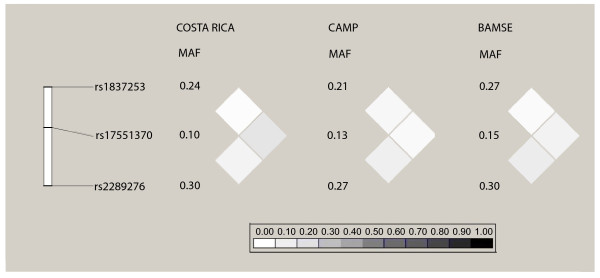
**LD plot of *TSLP *SNPs genotyped in Costa Rica, CAMP, and BAMSE**. Pairwise correlation structure for *TSLP *SNPs genotyped in all three cohorts.

**Figure 3:**
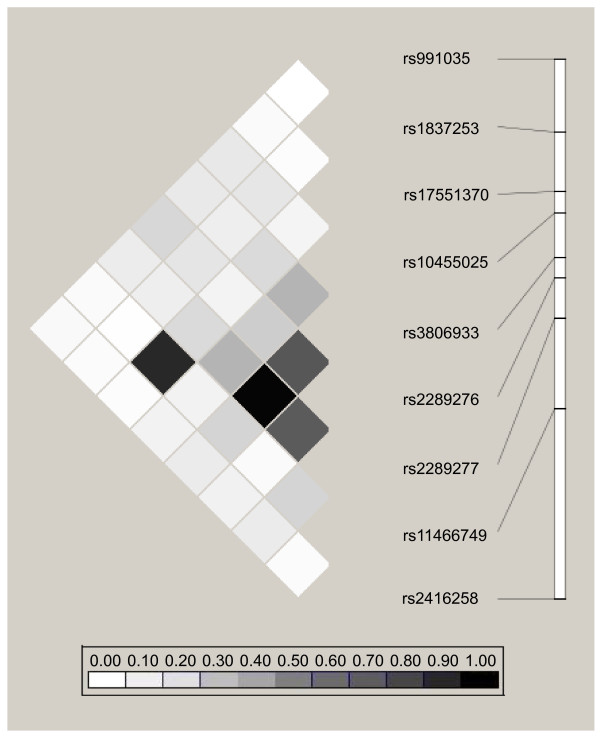
**LD plot of *TSLP *SNPs genotyped in Costa Rica**. Pairwise correlation structure for *TSLP *SNPs genotyped in Costa Rica

The linkage disequilibrium (LD) patterns and minor allele frequencies (MAF) for the *TSLP *SNPs genotyped in all three cohorts are shown in Figure 2. There were no major differences in MAFs and LD patterns among the SNPs between the cohorts. The LD patterns and MAFs for the nine *TSLP *SNPs genotyped in Costa Rica are shown in Figure 3. Consistent with our LD-tagging approach, LD was generally not high between the SNPs chosen for genotyping in Costa Rica.

The results for overall and sex-stratified association testing of *TSLP *SNP rs1837253 and AR in Costa Rica, CAMP, and BAMSE are shown in Table 4. The association between rs1837253 and AR in all children (in analyses not stratified by sex) was significant in CAMP (P value 0.003) but not in Costa Rica or BAMSE. In sex-stratified analysis, the T allele of SNP rs1837253 was associated with reduced odds for allergen-sensitized AR in boys in all three cohorts, with P values ranging from 0.04 to 0.004. Specifically, the minor allele of rs1837253 was undertransmitted in boys with AR and asthma as compared to boys with asthma alone in all three cohorts. The combined P value across cohorts met our criteria for significance after accounting for multiple testing, with Fisher's combined P value of 1.2 × 10^-4^. Odds ratios (ORs) for these associations ranged from 0.56 to 0.63. In contrast to the observed results in males, female-specific associations between rs1837253 and AR were inconsistent across cohorts.

**Table 4:** Association between *TSLP *SNP rs1837253 minor allele T* and allergic rhinitis in Costa Rica, CAMP, and BAMSE

	Costa Rica			CAMP			BAMSE		Combined
**Subjects**	**T:U**^**†**^	**OR**	**P value**	**T:U**	**OR**	**P value**	**OR**	**P value**	**Fisher's P value**

All children	104:122	0.85	0.18	50:81	0.62	0.003	0.72	0.089	0.003

Boys	59:93	0.63	0.004	32:55	0.58	0.007	0.56	0.04	1.2 × 10-4

Girls^	45:29	1.55	0.08	18:26	0.69	0.11	1.02	0.48	

*Allele frequency 0.24 in Costa Rica, 0.21 in CAMP, 0.27 in BAMSE

^†^T:U = ratio of transmitted to untransmitted alleles

^Fisher's combined P value not calculated because direction of effect inconsistent across females in these cohort.

The other *TSLP *SNPs and SNPs in *OX40L, IL7R *and *RXRα *were not significantly associated with AR phenotypes across cohorts (Table 5).

**Table 5:** Analysis of association between *TSLP, RXRa*, *IL7R, OX40L *SNPs and AR in Costa Rica, CAMP, and BAMSE

			Costa Rica				CAMP				BAMSE			
			
				P value				P value				P value		
								
SNP	Gene	Allele	Allele Frequency	All	Boys	Girls	Allele Frequency	All	Boys	Girls	Allele Frequency	All	Boys	Girls
rs1837253	*TSLP*	T	0.24	0.18	0.004	0.08	0.21	0.003	0.007	0.11	0.27	0.09	0.04	0.48

rs17551370	*TSLP*	A	0.10	0.77	0.95	0.71	0.13	0.31	0.32	0.42	0.15	0.03	0.25	0.03

rs2289276	*TSLP*	T	0.30	0.12	0.60	0.06	0.27	0.07	0.09	0.25	0.29	0.38	0.12	0.14

rs1234313	*OX40L*	A	0.38	0.26	0.10	0.81	0.32	0.007	0.03	0.048	0.34	0.04	0.03	0.29

rs10489267	*OX40L*	A	0.14	0.24	0.07	0.70	0.06	0.19	0.43	0.13	0.07	0.40	0.29	0.31

rs10489266	*OX40L*	G	0.08	0.16	0.13	0.69	0.11	0.36	0.27	0.42	0.10	0.25	0.21	0.48

rs1234315	*OX40L*	T	0.65	0.12	0.57	0.07	0.46	0.03	0.09	0.08	0.48	0.47	0.38	0.44

rs11185647	*RXRα*	A	0.41	0.53	0.76	0.54	0.29	0.19	0.046	0.25	0.26	0.42	0.18	0.07

rs12339187	*RXRα*	G	0.19	0.61	0.88	0.30	0.17	0.28	0.45	0.21	0.17	0.12	0.04	0.36

rs11185659	*RXRα*	T	0.22	0.74	0.80	0.39	0.20	0.43	0.21	0.22	0.21	0.05	0.02	0.49

rs10881582	*RXRα*	A	0.34	0.34	0.75	0.25	0.23	0.42	0.45	0.33	0.21	0.17	0.24	0.02

rs1494555	*IL7R*	G	0.47	0.74	0.49	0.77	0.34	0.38	0.43	0.20	0.26	0.04	0.13	0.08

rs6897932	*IL7R*	T	0.16	0.31	0.90	0.08	0.24	0.09	0.16	0.18	0.32	0.04	0.02	0.42

rs10063294	*IL7R*	G	0.60	0.85	0.80	0.55	0.48	0.36	0.36	0.13	0.42	0.05	0.11	0.13

rs2194225	*IL7R*	C	0.37	0.26	0.60	0.25	0.42	0.22	0.14	0.45	0.42	0.45	0.20	0.17

Tests for gene by gene interactions showed twelve nominally significant interactions between SNPs in all gene pair combinations of *TSLP, OX40L, RXRα *and *IL7R *except for between *RXRα *and *IL7R *(Table 6). After accounting for 84 tests for interaction, none remained significant after correction for multiple comparisons (P value threshold for significance 0.05/84 interaction tests = 0.00060).

**Table 6:** SNP by SNP Interaction Test P values in Costa Rica

Gene		*TSLP*	*TSLP*	*TSLP*	*OX40L*	*OX40L*	*OX40L*	*OX40L*	*RXRα*	*RXRα*	*RXRα*	*RXRα*	*IL7R*	*IL7R*	*IL7R*	*IL7R*
	**SNP**	rs1837253	rs17551370	rs2289276	rs1234313	rs10489267	rs10489266	rs1234315	rs11185647	rs12339187	rs11185659	rs10881582	rs1494555	rs6897932	rs10063294	rs2194225

***TSLP***	rs1837253				NS*	NS	NS	NS	NS	NS	NS	NS	**0.00062**	**0.0049**	**0.0033**	NS

***TSLP***	rs17551370				NS	NS	NS	NS	**0.036**	NS	NS	NS	NS	NS	NS	NS

***TSLP***	rs2289276				NS	NS	**0.034**	NS	NS	NS	NS	NS	NS	**0.016**	NS	NS

***OX40L***	rs1234313								NS	**0.046**	**0.033**	**0.0040**	NS	NS	NS	NS

***OX40L***	rs10489267								**0.021**	NS	NS	NS	NS	**0.018**	NS	NS

***OX40L***	rs10489266								NS	NS	NS	NS	NS	NS	NS	**0.043**

***OX40L***	rs1234315								NS	NS	NS	NS	NS	NS	NS	NS

***RXRα***	rs11185647												NS	NS	NS	NS

***RXRα***	rs12339187												NS	NS	NS	NS

***RXRα***	rs11185659												NS	NS	NS	NS

***RXRα***	rs10881582												NS	NS	NS	NS

***IL7R***	rs1494555															

***IL7R***	rs6897932															

***IL7R***	rs10063294															

***IL7R***	rs2194225															

*NS = non-significant

## Discussion

This is the first study to examine genetic associations between SNPs in *TSLP *and AR. This is also the first study to concurrently examine associations between variants in multiple *TSLP*-related genes (*OX40L, IL7R, RXRα*) and AR. We found an inverse male-specific association between the T allele of SNP rs1837253 in *TSLP *and AR in three independent cohorts of children with asthma. As children with asthma are particularly vulnerable to develop and suffer morbidity from AR, our findings are of direct relevance to this population.

Our study contributes to a nascent literature on the role of TSLP in AR. Prior work on TSLP has focused on other allergic diseases [6,9-12]. That TSLP could play a role in AR is first suggested by our understanding of TSLP and its ability to drive Th2 dominant inflammation. Second, a limited number of *in vitro *and murine studies have reported increased *TSLP *expression in cell cultures and nasal epithelium from AR patients [13-16,19]. The significant association between a *TSLP *variant and AR that we observed across multiple large and distinct cohorts supports that TSLP plays a role in AR in humans, corroborates previous *in vitro *and murine studies, and supports our understanding of TSLP driving allergic inflammation.

We found male-specific associations between *TSLP *SNP rs1837253 and AR in children with asthma from the Costa Rica, CAMP, and BAMSE studies. This SNP was undertransmitted in boys with AR and asthma as compared to in boys with asthma alone. Although this SNP has previously been associated with asthma [11,34], the associations with AR that we found cannot be attributed to asthma alone since all subjects in our study had asthma. Our findings suggest an additional role for this SNP in the pathogenesis of AR.

Our results were more statistically significant in boys than in all children, despite reductions in power in the sex-stratified analysis. This suggests that the significant associations among males that we observed across the cohorts were not merely due to the greater number of boys in these cohorts. The gene for the TSLPR chain of the heterodimeric TSLP receptor has a sex chromosome location in humans (Xp22.3 and Yp11.3) [41], and this could partially explain a sex-specific mechanism for *TSLP*. Sex-dependent hormonal regulation of transcription is also possible. Sex-specific effects have been observed for *TSLP *[11] and for other genes [42-44].

Our study's findings are consistent with sex-specific epidemiological observations for AR. Male children become more sensitized to environmental allergens and have higher serum total IgE levels [45,46]. Nasal fluid allergen-specific IgE levels are higher in male than female patients with seasonal AR [47]. A sex-stratified analysis of inflammatory pathways using allergen-challenged CD4+ cells from AR patients showed higher expression signatures in males [47]. Sexual dimorphism has also been noted in genetic linkage and association studies of serum total IgE [38,48]. Our findings expand on previous reports of sexual dimorphism in allergic disease by identifying sex-specific effects of a genetic variant on AR.

Our laboratory previously reported a sex-specific association between rs2289276 and serum total IgE [38], and between rs1827253 and asthma [11]. rs2289276 is predicted to affect an exonic splicing enhancer, while rs1837253 is thought to disrupt a transcription factor binding site [49]. SNP rs1837253 is located 5.7 kb upstream of the *TSLP *transcription start site. A study by Harada et al. conducted in human bronchial epithelial suggested that a SNP in the *TSLP *promoter region could serve as a binding site for transcription activating protein (AP)-1, enhance AP-1 binding to regulatory elements, and lead to *TSLP*'s downstream effects [33]. Harada et al. implicated rs3806933 as the functional SNP; they did not study rs1837253, and rs1837253 is not in LD with rs3806933 (Figure 3). We did not find an association between rs3806933 and AR, nor between rs2289276 and AR. He et al. reported associations between rs1837253 and protection from asthma, atopic asthma, and airway hyperresponsiveness [34], and Hunninghake et al. reported sex-specific associations between rs1837253 and protection from asthma in boys. These studies corroborate rs1837253 as a SNP of interest with a potential functional role.

Recognizing that TSLP interacts with other proteins to affect Th2-driven inflammation, we implemented a comprehensive approach from the outset by also examining for genetic associations between AR and SNPs in *OX40L, IL7R*, and *RXRα*. We did not observe findings that were consistent across cohorts for individual SNP associations with AR. This may have been due to unexamined gene-by-environment interactions. Shamim et al. previously reported an association between two *IL7R *SNPs and inhalation allergy [50]. They did not perform replication analyses in independent populations, and we did not find associations between those SNPs and AR in our cohorts. It is thought that RXRα and RXRβ can influence transcription of *TSLP*, but neither has been previously studied in subjects with AR [23]. The *RXRα *SNPs we chose to genotype capture 96% of the HapMap SNPs with MAF ≥ 10% in *RXRα *and its 10 kb flanks in the CEU population with r^2 ^≥ 0.8, so our lack of findings for *RXRα *was unlikely due to inadequate genotypic coverage [32]. Interestingly, our tests for gene by gene interaction among *TSLP, OX40L, RXRa *and *IL7R *in Costa Rican subjects demonstrated nominally significant results between SNPs in all gene pairs, except for between *RXRα *and *IL7R*. Lack of gene by gene interaction between *RXRα *and *IL7R *would be biologically consistent with their physically disparate roles as transcriptional regulator of *TSLP *and receptor for TSLP, respectively. Further gene by gene analyses with larger sample size could overcome the power limitations we faced.

Our study has additional limitations. First, our findings do not elucidate a specific mechanism. SNP rs1837253 is not in LD with other HapMap SNPs and has the potential to represent a functional SNP itself. Our work provides a specific direction for functional studies that could focus on transcriptional regulators of *TSLP*. Second, some of our findings in Costa Ricans may be mainly applicable to them and certain Hispanic subgroups. However, we replicated our main finding in CAMP and BAMSE, and previous findings in Costa Rica have been applicable to children of other ethnicities [51,52]. Family-based testing is also robust against population stratification and population admixture [36]. Lastly, we focused on AR in children with asthma, and it is possible that distinct associations could be found if we examined cohorts with AR only. However, our findings are relevant to a group of children at high risk for AR.

## Conclusions

In summary, we found that the T allele of *TSLP *SNP rs1837253 was associated with reduced odds for AR in three independent cohorts of children with asthma. The association was sex-specific, as it was significant in males but not females. Our work highlights that *TSLP *likely plays a role in the pathogenesis of AR in children with asthma.

## Competing interests

The authors declare that they have no competing interests.

## Authors' contributions

SB contributed to the study design, analyzing the data, and writing the manuscript. EM contributed to analyzing the data and manuscript editing. JBW contributed to analyzing the data and manuscript editing. MG contributed to analyzing the data and manuscript editing. MS contributed to patient recruitment and manuscript editing. LA contributed to patient recruitment and manuscript editing. JLS contributed to analyzing the data and manuscript editing. GH contributed to the study design and manuscript editing. MW contributed to patient recruitment and manuscript editing. GP contributed to patient recruitment and manuscript editing. GTO contributed to patient recruitment and manuscript editing. SW contributed to patient recruitment and manuscript editing. JCC contributed to the study design, patient recruitment and writing the manuscript. All authors read and approved the final manuscript.
